# ROSES‐S: Statement from the World Health Organization on the reporting of seroepidemiologic studies for SARS‐CoV‐2

**DOI:** 10.1111/irv.12870

**Published:** 2021-06-26

**Authors:** World Health Organization Seroepidemiology Technical Working Group

**Keywords:** emerging, infectious diseases, SARS‐CoV‐2, seroepidemiologic Studies

## Abstract

Well‐designed population‐based seroepidemiologic studies can be used to refine estimates of infection severity and transmission, and are therefore an important component of epidemic surveillance. However, the interpretation of the results of seroepidemiologic studies for SARS‐CoV‐2 has been hampered to date principally by heterogeneity in the quality of the reporting of the results of the study and a lack of standardized methods and reporting. We provide here the ROSES‐S: Reporting of Seroepidemiologic studies—SARS‐CoV‐2. This is an updated checklist of 22 items that should be included in the reporting of all SARS‐CoV‐2 seroepidemiologic studies, irrespective of study design.

## 1 INTRODUCTION

### 1.1 Main text

When a new pathogen such as SARS‐CoV‐2 emerges, there are critical questions to answer on the level of pre‐existing immunity; the full spectrum of illness, including the proportion of infections that are asymptomatic, the proportion that require medical care, and the proportion that cause death; and the level of infection in specific populations. Seroepidemiologic studies, which estimate the prevalence of individuals with antibodies against a pathogen, provide information to answer these questions and are, hence, an important component of epidemic surveillance.

Understanding the proportion of the population who remain susceptible to infection can be used to inform disease modeling, forecasting, and the optimal implementation of public health and social measures. As initial surveillance in an outbreak of an infectious disease primarily focuses on individuals who require hospitalization, mild and asymptomatic infections are not typically captured. For infectious diseases, such as COVID‐19, with a clinical presentation that extends beyond those who require hospitalization to include asymptomatic and mild infections, seroepidemiologic studies are central to capture infection irrespective of disease severity. The results of well‐designed population‐based seroepidemiologic studies can then be used to refine estimates of infection severity and transmission.

Following the emergence of SARS‐CoV‐2, the virus that causes COVID‐19, serologic assays have been rapidly developed to reliably detect anti‐SARS‐CoV‐2 antibodies in serum samples, including assays that are now available commercially.^1^ The predictive value of these immunoassays is influenced by prevalence of infection.^2^, ^3^, ^4^ Sensitivity and specificity are influenced by the assay format, differences in the antibody isotype targeted (eg, IgM, IgG, IgM + IgG, IgA, and/or total antibodies), the viral antigen used^2^, ^5^ the time from infection to time of biological sampling,^4^ and the severity of disease.^6^ Cross‐reactive antibodies with seasonal and zoonotic coronaviruses have been observed for some assays, and their prevalence may differ depending on the population tested.

Several systematic reviews have now been conducted of the initial pre‐print or peer‐reviewed SARS‐CoV‐2 seroepidemiologic studies.^7^, ^8^, ^9^ These reviews have found overall seroprevalence to be low, although these reviews reflect seroprevalence results before August 2020. They showed variation by country and by region, with higher seropositivity reported in high‐risk groups (eg, healthcare workers) and populations that have experienced widespread community transmission. Importantly, they have also highlighted heterogeneity in the quality of many of the initial seroprevalence studies, derived principally from the quality of the reporting of the results of the study, as has been shown for other diseases.^10^ Many fail to report on the validation of the immunoassay used for specific application to the study, or provide sufficient information as to how the immunoassay was validated, or report on whether seroprevalence estimates were corrected for sampling biases or immunoassay performance. In the absence of the use of the same assay used with standardized cutoffs and confirmatory testing algorithms, these factors hamper the interpretation of the study results, as well as efforts to pool study results to understand population seroprevalence at a country, regional, or global level.

In 2016, the Reporting of Seroepidemiologic studies for influenza (ROSES‐I statement) was published by the Consortium for the Standardization of Influenza Seroepidemiology (CONSISE) in an effort to improve the quality and transparency of reporting of influenza seroepidemiologic studies and facilitate the assessment of the validity and generalizability of published results.^11^ This statement was developed as an extension of the STROBE statement^12^ and identified 22 items that should be included in the results of published seroepidemiologic studies. As such, this checklist is also recommended to be used to guide the design and implementation of seroepidemiologic studies.

Here, we provide in Table 1 a modified version of the statement—(ROSES‐S: Reporting of Seroepidemiologic studies—SARS‐CoV‐2)—for application to seroepidemiologic studies conducted during the ongoing COVID‐19 pandemic. This covers the elements that should be reported in all SARS‐CoV‐2 seroepidemiologic studies, irrespective of study design.

**TABLE 1:** Checklist for the reporting of SARS‐CoV‐2 seroepidemiologic studies

Item number	Item	ROSES‐I description (Horby P, 2016)	ROSES‐I description for SARS‐CoV‐2
Title, abstract and introduction			
1	Title and abstract	The term “seroepidemiologic,” “seroepidemiology,” “seroprevalence,” or “seroincidence” should be applied to the study in the title or abstract, and the medical subject heading “Seroepidemiologic Studies” be used when the report is of a population‐based serological survey.	The term “seroepidemiologic,” “seroepidemiology,” “seroprevalence,” or “seroincidence” should be applied to the study in the title and abstract, and the medical subject heading “Seroepidemiologic Studies” be used when the report is of a population‐based serological survey. Provide a structured summary including, as applicable: objectives; population level (ie, national, regional, local), study design, study period, eligibility criteria of study participants, sampling dates and method, sample size, laboratory methods (assay used), results: seroprevalence and 95% CI, study limitations, conclusions and implications of key findings.
2	Introduction	State what is known about the kinetics of antibody rise, decay, and persistence following infection for the particular virus being studied and the justification for threshold antibody titers or changes in titers used to define evidence of infection. State what is known about the sensitivity and specificity of the antibody detection assay being used.	State what is known about the kinetics of antibody rise, decay, and persistence following SARS‐CoV‐2 infection, in the particular study setting/population, if possible. State which SARS‐CoV‐2 viruses are circulating, including any variants State what is known about the sensitivity and specificity of the antibody detection assay being used.
3		State specific objectives, including any prespecified hypotheses.	State specific objectives, including any prespecified hypotheses.
Epidemiological methods			
4	Study design	State which specific seroepidemiologic study design was chosen and why.	State which specific seroepidemiologic study design was chosen and why.
5	Setting	Describe the timing of the biological sampling in relation to the disease epidemiology in the study population (the beginning, peak, and end of virus transmission). Where known, describe the timing of biological sampling in individuals in relation to disease onset and to exposures of interest. State the interval between sequential biological samples (serial cross‐sectional or longitudinal studies), or specify whether only a single sample was collected (cross‐sectional study).	Describe the setting, locations, and sampling frame, including periods of recruitment, exposure, follow‐up, and data collection. Describe the timing of the biological sampling in relation to the disease epidemiology in the study population (the beginning, peak, and end of virus transmission). Describe any vaccination efforts that have been undertaken. Where known, describe the timing of biological sampling in individuals in relation to disease onset and to exposures of interest. State the interval between sequential biological samples (serial cross‐sectional or longitudinal studies), or specify whether only a single sample was collected (cross‐sectional study).
6	Participants	For case‐ascertained transmission studies, describe the method of case ascertainment and criteria for defining a “case.” For household‐ or institution‐based transmission studies, describe the definition of a household or the institution. For outbreak investigations involving serologic sampling, describe the setting in which the cases were identified, for example, village/residential setting, occupational workplace. To aid the interpretation of seroepidemiologic studies of novel influenza A virus subtypes, the results from exposed populations should be compared with the results from unexposed populations. Efforts to validate the assay in virologically confirmed cases should be reported.	For case‐ascertained transmission studies, describe the method of case ascertainment and criteria for defining a “case.” Describe methods of follow‐up. For household‐ or institution‐based transmission studies, describe the definition of a household or the institution. Describe methods of follow‐up. For outbreak investigations involving serologic sampling, describe the setting in which the cases were identified, for example, village/residential setting, occupational workplace. Describe methods of follow‐up. For a cohort study, give the eligibility criteria, and the sources and methods of sampling of participants. Describe methods of follow‐up. For a case‐control study, give the eligibility criteria, and the sources and methods of case ascertainment and control selection. Give the rationale for the choice of cases and controls. For matched studies, give matching criteria and the number of controls per case. For a cross‐sectional study, give the eligibility criteria, and the sources and methods of selection of participants
7	Variables	Describe the potential for immunization (specify vaccine and timing of vaccination in relationship to collection of serum), if applicable, to affect the outcome measures. Describe any known or potential immunological cross‐reactivity that may bias the outcome measures. Describe illness definitions and methods for ascertaining the presence or absence of clinical illness in subjects.	Clearly define all outcomes, exposures, predictors, potential confounders, and effect modifiers. The median age and range for each exposure group should be reported. Describe the vaccination status of participants (specify vaccination status, vaccine manufacturer, number of doses, and timing of vaccination in relationship to collection of serum), if applicable, to affect the outcome measures. If relevant, describe measures taken to identify and record immunization history. Describe any known or potential immunological cross‐reactivity that may bias the outcome measures. Describe illness definitions and methods for ascertaining the presence or absence of clinical illness in subjects.
8	Data sources/measurement biases	If relevant, describe measures taken to identify and record immunization history.	For each variable of interest, give sources of data and details of methods of assessment (measurement). Describe comparability of assessment methods if there is more than one group. Give information separately for cases and controls in case‐control studies and, if applicable, for exposed and unexposed groups in cohort and cross‐sectional studies).
9	Bias	If relevant, describe efforts to control for the potential effect of immunization on estimates of outcomes.	Describe any efforts to address potential sources of bias.
10	Study size	Describe the baseline estimated seroprevalence at given antibody titers or incidence of infection and cite published literature to support these estimates.	Describe the baseline estimated seroprevalence or incidence of infection and cite published literature to support these estimates. Explain the steps that led to the final sample size. Report the numbers of individuals at each stage of the study—the numbers potentially eligible, examined for eligibility, confirmed eligible, included in the study, completing follow‐up, and analyzed.
11	Quantitative variables	Describe the serological assay's limit of detection and how this limit is defined or calculated. Describe how samples with a result below or on the borderline of the limit were handled in the analysis. Describe and justify the titer or other result used to define “seropositivity,” or the antibody titer change or change in other assay result used to define “seroconversion.” Avoid the term “seroconversion” unless referring to change from undetectable to detectable antibody level. Otherwise report the fold‐rise in titer. Avoid the term “infection” but report “seroprevalence at a titer of ….”. If statements or inferences are made about protection from infection, describe what is known about the correlation between the assay results and protection from infection and illness.	Explain how quantitative variables were handled in the analyses. If applicable, describe which groupings were chosen and why. Describe the serological assay's limit of detection and how this limit is defined or calculated. Describe how samples with a result below or on the borderline of the limit were handled in the analysis. Define “seropositivity,” or the antibody titer change or change in other assay result used to define “seroconversion.” Avoid the term “seroconversion” unless referring to change from undetectable to detectable antibody level. Avoid the term “infection” but report “seroprevalence at a titer of ….”.
12	Statistical methods	If relevant, state how the non‐independence of data was managed. If relevant, report methods used to account for the probability of seropositivity or seroconversion if infected, and to account for decay in antibody titers over time.	Describe all statistical methods, including those used to control for confounding. Describe any methods used to examine subgroups and interactions. Describe all methods used to address sampling and selection biases (eg, weighting results, multilevel regression and post‐stratification). Explain how missing data were addressed. For a cohort study, explain how loss to follow‐up was addressed, if applicable. For a case‐control study, explain how variables on which cases and controls were matched, if applicable. For a cross‐sectional study, describe analytical methods taking account of sampling strategy, if applicable. Describe any sensitivity analyses. If relevant, report methods used to account for adjustment for assay performance (sensitivity and specificity), the probability of seropositivity or seroconversion if infected, and to account for decay in antibody titers over time.
Laboratory methods			
13	Sample type and handling	Describe the sample type—serum or plasma. If plasma is used, specify the anticoagulant used (heparin, sodium citrate, EDTA, etc). Describe the specimen storage conditions (4°C, −20°C, −80°C). If frozen prior to the analysis, describe the time to freezing and the number of freeze/thaw cycles prior to testing.	Describe the sample type—whole blood, dried blood, serum or plasma. If plasma is used, specify the anticoagulant used (heparin, sodium citrate, EDTA, etc). Describe the specimen storage conditions (4°C, −20°C, −80°C). If frozen prior to the analysis, describe the time to freezing and the number of freeze/thaw cycles prior to testing.
	Serological assays	Specify the assay type (eg, receptor‐binding inhibition; virus neutralization/microneutralization; ELISA; other) and methods used to determine the endpoint titer. Reference a previously published, CONSISE consensus serologic assay or WHO protocol if used, and any modifications of the protocol. If a previously published protocol is not used, provide full details in supplementary materials. State what is known about the determinants of the variability of the antibody detection assay being used. Specify the antigen(s) used in the assay, including virus strain name, subtype, lineage or clade, with standardized nomenclature and reference; specify whether live virus or inactivated virus was used (where applicable). Report if antigen(s) from potentially cross‐reactive pathogens/strains were used in order to identify cross‐reactivity, and specify which antigen was used, including virus name, subtype, strain, lineage and clade, with standardized nomenclature and reference. If red blood cells were used for a hemagglutinin inhibition assay, specify the animal species from which they were obtained and concentration (v/v) used. Describe positive and negative controls used. Describe starting and end dilutions. Specify laboratory biosafety conditions. Specify whether replication was performed, and if so, the acceptable replication parameters. Specify whether a confirmatory assay was performed and all specifics of this assay, at the same level of detail. Specify international standards used, if appropriate	Wherever possible, use defined and standardized methods that have been established in more than one laboratory, and that ideally are commercially available in more than one country. Avoid laboratory‐level formulations if standardized formulations are available for the same analytical targets. Specify the testing algorithm (if more than one test used) and assay type (eg, virus neutralization/microneutralization/surrogate neutralization; ELISA; LFIA; CLIA; other) and readout used to determine the endpoint titer. Reference a previously published protocol, if used, and any modifications of the protocol. If a previously published protocol was not used, provide full details in supplementary materials. For in‐house assays, include a description of the assay format (e.g., direct or indirect immunoassay) as well as description of cutoff determination and which antibody isotype is targeted, and reference previously published validation data. State what is known about the determinants of the variability of the antibody detection assay being used. Specify the antigen(s) and antibody isotope target used, with standardized nomenclature and reference; specify whether live virus or pseudo virus was used (where applicable). Describe how the cutoff was established. If viral antigen produced in‐house is used, specify sequence, expression system (bacteria or mammalian cells). Specify reactivity with other coronavirus antigens (MERS‐CoV, SARS‐CoV, seasonal CoVs) in the same population. Describe positive and negative controls used. Specify international standards used, if appropriate. Describe starting and end dilutions. Specify laboratory biosafety conditions. Specify whether replication was performed, and if so, the acceptable replication parameters. Specify whether a confirmatory assay was performed and all specifics of this assay, at the same level of detail.
Results			
13	Participants	Report the numbers of individuals at each stage of the study—the numbers potentially eligible, examined for eligibility, confirmed eligible, included in the study, completing follow‐up, and analyzed. Give reasons for non‐participation at each stage. Consider use of a flow diagram.	Report the numbers of individuals at each stage of the study—the numbers potentially eligible, examined for eligibility, confirmed eligible, included in the study, completing follow‐up, and analyzed. Give reasons for non‐participation at each stage. Consider use of a flow diagram.
14	Descriptive data	Give characteristics of study participants (eg, demographic, clinical, social) and information on exposures and potential risk factors. Indicate the number of participants with missing data for each variable of interest. Cohort study—summarize follow‐up time (eg, average and total amount).	Give characteristics of study participants (eg, demographic, clinical, social) and information on exposures and potential risk factors for all participants, not solely stratified by outcome status. Indicate the number of participants with missing data for each variable of interest. For a cohort study, detail follow‐up time (eg, average and total amount).
15	Outcome data	Cohort study—report the numbers of outcome events or summary measures over time. Case–control study—report the numbers in each exposure category, or summary measures of exposure. Cross‐sectional study—report the numbers of outcome events or summary measures.	For a cohort study, report the numbers of outcome events or summary measures over time. For a case‐control study, report the numbers in each exposure category, or summary measures of exposure. For a cross‐sectional study, report the numbers of outcome events or summary measures.
16	Main result	Report unadjusted estimates of distribution of titers by age group. Report methods to standardize the results from the study sample to the target population.	Report unadjusted estimates of distribution of seropositivity by age group. Report methods to standardize the results from the study sample to the target population.
17	Other analyses	Report other analyses performed—analyses of subgroups and interactions, and sensitivity analyses.	Report other analyses performed—analyses of subgroups and interactions, and sensitivity analyses.
Discussion			
18	Key results	Summarize key results with reference to study objectives.	Summarize key results with reference to study objectives.
19	Limitations	Discuss limitations and strengths of the study.	Discuss limitations and strengths of the study.
20	Interpretation	Discuss the interpretation of the results in the context of known or potential cross‐reactivity.	Discuss the interpretation of the results in the context of known or potential cross‐reactivity, assay performance and other sources of bias.
21	Generalizability	Discuss the generalizability (external validity) of the study results.	Discuss the generalizability (external validity) of the study results.
Other information			
22	Ethics approval	Specify if institutional review board approval was received; if not, specify reason (eg, public health outbreak response/non‐research designation).	Specify if institutional review board approval was received; if not, specify reason (eg, public health outbreak response/non‐research designation).

In addition, WHO, in collaboration with a range of technical partners, has developed seroepidemiologic protocol templates to answer a series of key public health questions, known as the Unity studies, that are available on the WHO website (https://www.who.int/emergencies/diseases/novel‐coronavirus‐2019/technical‐guidance/early‐investigations). These protocols provide standardized epidemiological and serological methodology that will better facilitate comparisons between studies (in time and place) and secondary data analyses. WHO has also established the Solidarity II global collaboration of public health agencies and academic institutions (https://www.who.int/emergencies/diseases/novel‐coronavirus‐2019/global‐research‐on‐novel‐coronavirus‐2019‐ncov/solidarity‐2‐global‐serologic‐study‐for‐covid‐19). This forum allows the sharing of well‐characterized panels of sera to enable standardization of serologic assays worldwide and access to high‐quality antigen specifically for assays to conduct serologic surveys. Solidarity II is also developing a standardized serology assay for collaborators who wish to use a global standard assay and methodologies for laboratories around the world to develop their own serologic assays. It facilitates the sharing of laboratory protocols for serologic assays for the purposes of serology surveys and study protocols, such as the Unity studies.

Understanding population seroprevalence over time is important for informing public health decisions made by health authorities and policy makers. Conducting and reporting studies that are aligned with this ROSES‐S checklist will allow for more refined epidemic modeling, outbreak responses, and public health and social measures, as well as more complete secondary data analyses.

## CONFLICT OF INTEREST

The authors have no conflict of interest to declare.

## PATIENT CONSENT STATEMENT

Patient consent was not applicable to this article as no patients were involved in the current study.

## PERMISSION TO REPRODUCE MATERIAL FROM OTHER SOURCES

Permission to reproduce material from other sources is not applicable to this article.

## AUTHOR CONTRIBUTIONS

**Maria van Kerkhove:** Conceptualization (equal); Methodology (equal); Writing‐review & editing (equal). **Rebecca Grant:** Methodology (equal); Writing‐original draft (equal). **Lorenzo** **Subissi:** Validation (equal); Writing‐review & editing. **Marta** **Valenciano:** Validation (equal); Writing‐review & editing. **Ketevan Glonti:** Validation (equal); Writing‐review & editing. **Isabel** **Bergeri:** Validation (equal); Writing‐review & editing. **Polina Brangel:** Validation (equal); Writing‐review & editing. **Olivier Le** **Polain:** Validation (equal); Writing‐review & editing. **Hannah Lewis:** Validation (equal); Writing‐review & editing. **Anthony** **Nardone:** Validation (equal); Writing‐review & editing. **Richard** **Pebody:** Conceptualization; Methodology; Validation (equal); Writing‐review & editing. **Tasnim Azim:** Validation (equal); Writing‐review & editing. **Pushpa Wijesinghe:** Validation (equal); Writing‐review & editing. **Lubna Al Ariqi:** Validation (equal); Writing‐review & editing. **Linh‐Vi Le:** Validation (equal); Writing‐review & editing. **Joseph** **Okeibunor:** Validation (equal); Writing‐review & editing. **Andrea** **Vicari:** Validation (equal); Writing‐review & editing. **Amen Ben** **Hamida:** Validation (equal); Writing‐review & editing. **Venkatachalam Udhayakumar:** Validation (equal); Writing‐review & editing. **Kathleen Gallagher:** Validation (equal); Writing‐review & editing. **Vincent Richard:** Validation (equal); Writing‐review & editing. **Rahul Arora:** Validation (equal); Writing‐review & editing. **Niklas Bobrovitz:** Validation (equal); Writing‐review & editing. **Maria** **Zambon:** Validation (equal); Writing‐review & editing. **Christian** **Drosten:** Validation (equal); Writing‐review & editing. **Marion Koopmans:** Validation (equal); Writing‐review & editing. **Malik JS Peiris:** Validation; Writing‐review & editing.

## 2 ETHICS STATEMENT

Ethics approval was not applicable to this article.

### PEER REVIEW

The peer review history for this article is available at https://publons.com/publon/10.1111/irv.12870.

## Data Availability

Data sharing is not applicable to this article as no datasets were generated or analyzed during the current study.

## Appendix

WHO HQ: Maria D Van Kerkhove, Rebecca Grant, Lorenzo Subissi, Marta Valenciano, Ketevan Glonti, Isabel Bergeri, Polina Brangel, Olivier Le Polain, Hannah Lewis, Anthony Nardone. WHO REGIONAL OFFICES: Richard Pebody, Tasnim Azim, Pushpa Wijesinghe, Lubna Al Ariqi, Linh‐Vi Le, Joseph Okeibunor, Andrea Vicari. EXTERNAL PARTNERS: Amen Ben Hamida (United States Centers for Disease Control and Prevention), Venkatachalam Udhayakumar (United States Centers for Disease Control and Prevention), Kathleen Gallagher (United States Centers for Disease Control and Prevention), Vincent Richard (Institut Pasteur), Rahul Arora (University of Oxford), Niklas Bobrovitz (University of Oxford). Maria Zambon (Public Health England), Christian Drosten (Charité), Marion Koopmans (Erasmus University Medical Center), Malik Peiris (Hong Kong University).
